# A finite element analysis of sacroiliac joint displacements and ligament strains in response to three manipulations

**DOI:** 10.1186/s12891-020-03735-y

**Published:** 2020-10-28

**Authors:** Zhun Xu, Yikai Li, Shaoqun Zhang, Liqing Liao, Kai Wu, Ziyu Feng, Dan Li

**Affiliations:** 1 School of Traditional Chinese Medicine, Southern Medical University, No. 1838, Guangzhou Avenue North, BaiYun District, Guangzhou, 510515 Guangdong Province China; 2 Department of Spine Surgery, The First Affiliated Hospital of University of South China, Hengyang, 421000 Hunan Province China; 3 ShenZhen Traditional Chinese Medicine Hospital, Shenzhen, Guangdong PR China

**Keywords:** Manipulation, Sacroiliac joint, Displacement, Ligament strain, Finite element analysis

## Abstract

**Background:**

Clinical studies have found that manipulations have a good clinical effect on sacroiliac joint (SIJ) pain without specific causes. However, the specific mechanisms underlying the effect of manipulations are still unclear. The purpose of this study was to investigate the effects of three common manipulations on the stresses and displacements of the normal SIJ and the strains of the surrounding ligaments.

**Methods:**

A three-dimensional finite element model of the pelvis-femur was developed. The manipulations of hip and knee flexion (MHKF), oblique pulling (MOP), and lower limb hyperextension (MLLH) were simulated. The stresses and displacements of the SIJ and the strains of the surrounding ligaments were analyzed during the three manipulations.

**Results:**

MOP produced the highest stress on the left SIJ, at 6.6 MPa, while MHKF produced the lowest stress on the right SIJ, at 1.5 MPa. The displacements of the SIJ were all less than 1 mm during the three manipulations. The three manipulations caused different degrees of ligament strain around the SIJ, and MOP produced the greatest straining of the ligaments.

**Conclusion:**

The three manipulations all produced small displacements of the SIJ and different degrees of ligament strains, which might be the mechanism through which they relieve SIJ pain. MOP produced the largest displacement and the greatest ligament strains.

## Background

The sacroiliac joint (SIJ) is the largest axial joint in the human body; it connects the spine and the lower limbs and transmits the weight of the upper body to the pelvis and lower limbs [1–3]. The SIJ is composed of an anterior synovial part and a tightly connected ligament part at the rear [4, 5]. The sacrum is wedge-shaped, tilted from top to bottom and with a concave surface that is closely inserted into the convex surface of the ilium [6, 7]. It includes some strong intrinsic and extrinsic ligaments [8, 9]. Due to its special anatomical structure, the SIJ is very stable [10–12]. However, certain bone or soft tissue lesions can cause joint instability, which subsequently induces SIJ pain. Recent studies have found that SIJ diseases can also cause low back pain and account for approximately 14.5–22.5% of cases [13].

The causes of SIJ pain include pathological bone destruction, traumatic fracture and dislocation, and pain without specific causes [5]. Commonly, abnormal gait, heavy physical exertion, leg length discrepancy, inflammation, scoliosis, and lumbar fusion surgery with fixation of the sacrum may be factors related to SIJ pain without specific causes [14]. The mechanism may include the following processes: Pathogenic factors acting on the auricular surface of the sacrum and ilium may cause injury to the ligaments or muscles around the SIJ, which will result in slight movement of the SIJ, making the joints difficult to reset. The mechanical environment of the joints may ultimately be imbalanced, and the soft tissues will be damaged. This condition is clinically referred to as SIJ subluxation [4]. Pathological bone destruction and traumatic fracture and dislocation require surgery [15–18], while SIJ pain without a specific cause is usually treated with manipulations [19, 20].

There are several common manipulations, including manipulation of hip and knee flexion (MHKF), manipulation of oblique pulling (MOP), and manipulation of lower limb hyperextension (MLLH). A large number of studies have reported that manipulations have a good effect on the treatment of SIJ subluxation [19–21]. Different types of SIJ subluxation require different manipulations [22, 23]. Some authors have suggested that the manipulations reduce pain by pulling the subluxated SIJ back into place [23–25]. Others have suggested that the SIJ is very stable and that the pain relief is the result of relieving the spasms of the ligaments and muscles around the SIJ [26, 27]. However, the mechanisms underlying the effect of manipulations on SIJ subluxation are not clear at present. Is it possible to pull the subluxated SIJ back into place with manipulations? Can manipulations cause ligament strain around the joints? None of these issues have been studied. In this study, the normal pelvic-femur finite element model is used to investigate the specific mechanisms underlying the effects of the three manipulations on the SIJ and its surrounding ligaments.

## Methods

### Model construction

A 3D finite element model of the SIJ was developed. Three-dimensional models of the sacrum, ilia and femurs were reconstructed from the computed tomography (CT) images of a healthy male volunteer (34 years old, 170 cm in height, and 65 kg in weight) using Mimics 20.0 (Materialise Company, Leuven, Belgium), and the cortical and cancellous regions of the bones were distinguished. Axial slices 0.5-mm thick spanning the entire pelvis were selected for model construction. All surface models were meshed using Geomagic 2013 (Raindrop Company, Marble Hill, USA). The SIJ is composed of cartilage and the end-plate of the sacrum and the ilia, with their surrounding ligaments. The cartilage was reconstructed with a uniform thickness; the regions of the articular surfaces were based on CT images, and the thicknesses of the cartilage were acquired from the literature. The sacral and iliac cartilages had thicknesses of 2 mm and 1 mm, respectively. The bone end-plate thicknesses of the sacral and iliac parts of the cartilage were assumed to be 0.23 mm and 0.36 mm, respectively. The gap between the two cartilages was set at 0.3 mm [12]. The material properties chosen from previous studies [12, 28] are summarized in Table 1.

**Table 1:** Material properties of the sacrum, ilium, femur, pubic symphysis and endplate

		Young’s modulus (MPa)	Poisson’s ratio
Sacrum	Cortical	12,000	0.3
	Cancellous	100	0.2
Ilium	Cortical	12,000	0.3
	Cancellous	100	0.2
Femur	Cortical	15,000	0.3
	Cancellous	100	0.2
Pubic symphysis		5	0.45
Articular cartilage		100	0.3
Endplate		1000	0.4

**Fig. 1:**
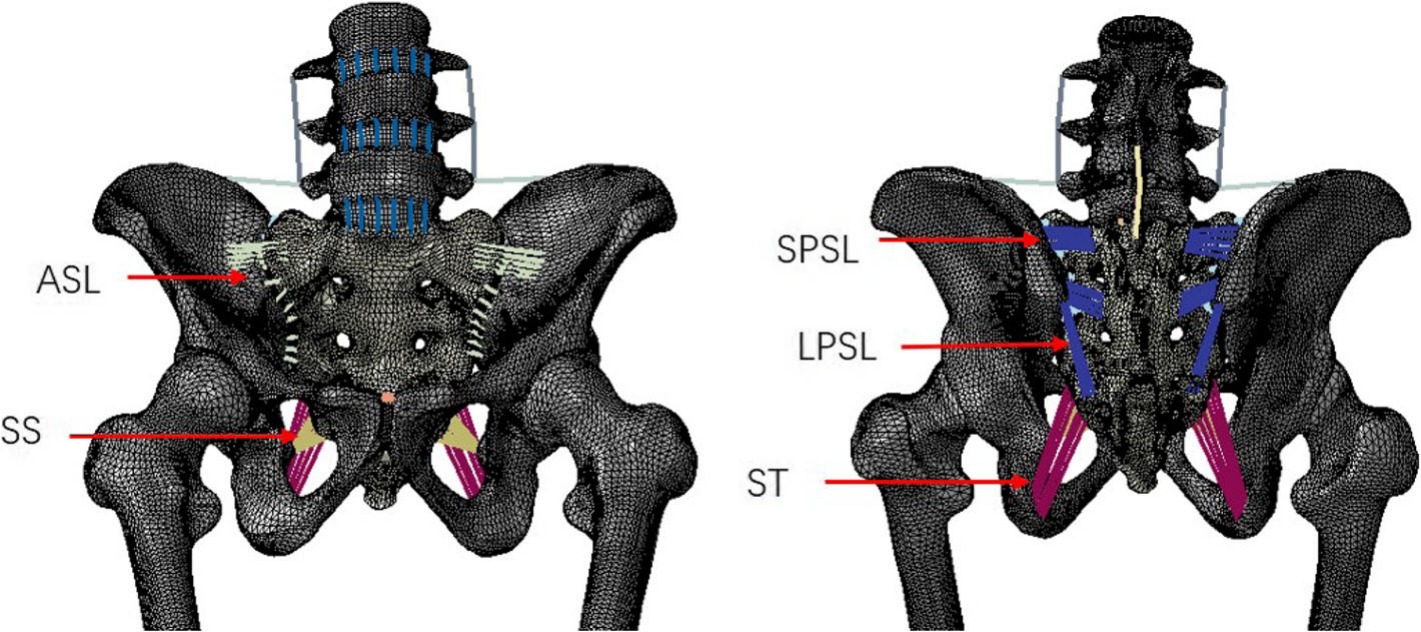
Ventral (left) and Dorsal (right) views of the finite element model. Ligaments are represented in color lines, with red arrows identifying each ligament complex (note the interosseous sacroiliac ligament is not visible in anterior-posterior views). ASL indicates anterior sacroiliac ligament; LPSL, long posterior sacroiliac ligament; SPSL, short posterior sacroiliac ligament; SS, sacrospinous ligament; ST, sacrotuberous ligament

The anterior sacroiliac ligament (ASL), long posterior sacroiliac ligament (LPSL), short posterior sacroiliac ligament (SPSL), interosseous sacroiliac ligament (ISL), sacrospinous ligament (SS), and sacrotuberous ligament (ST) complexes were modelled as 3D tension-only truss elements. The attachment regions were chosen according to the literature [12]. Two fresh cadaver dissections were used to observe the ligaments’ positions and orientations. The ASL was made up of numerous thin bands that spanned the ventral surface of the SIJ, connecting the lateral aspect of the sacrum to the margin of the auricular surface of the ilium. The LPSL extended from the posterior superior iliac spine to the third and fourth transverse tubercles of the back of the sacrum. The SPSL lay deep to the LPSL and consisted of large fibres attaching the lateral aspect of the dorsal sacral surface to the tuberosity of the ilium. The ISL lay in the intra-articular space and was composed of a series of short, strong fibres connecting the tuberosities of the sacrum and ilium. The SS was a thin triangular ligament that connected the ischial spine to the lateral border of the sacrum. The ST was behind the sacrospinous ligament, which attached the ischial tuberosity to the lateral border of the sacrum. The material properties of each ligament were obtained from the literature [28]. In total, the pelvic-femur model contained 727,474 elements and 275,399 nodes. Figure 1 shows the intact model with ligamentous attachments.

**Fig. 2:**
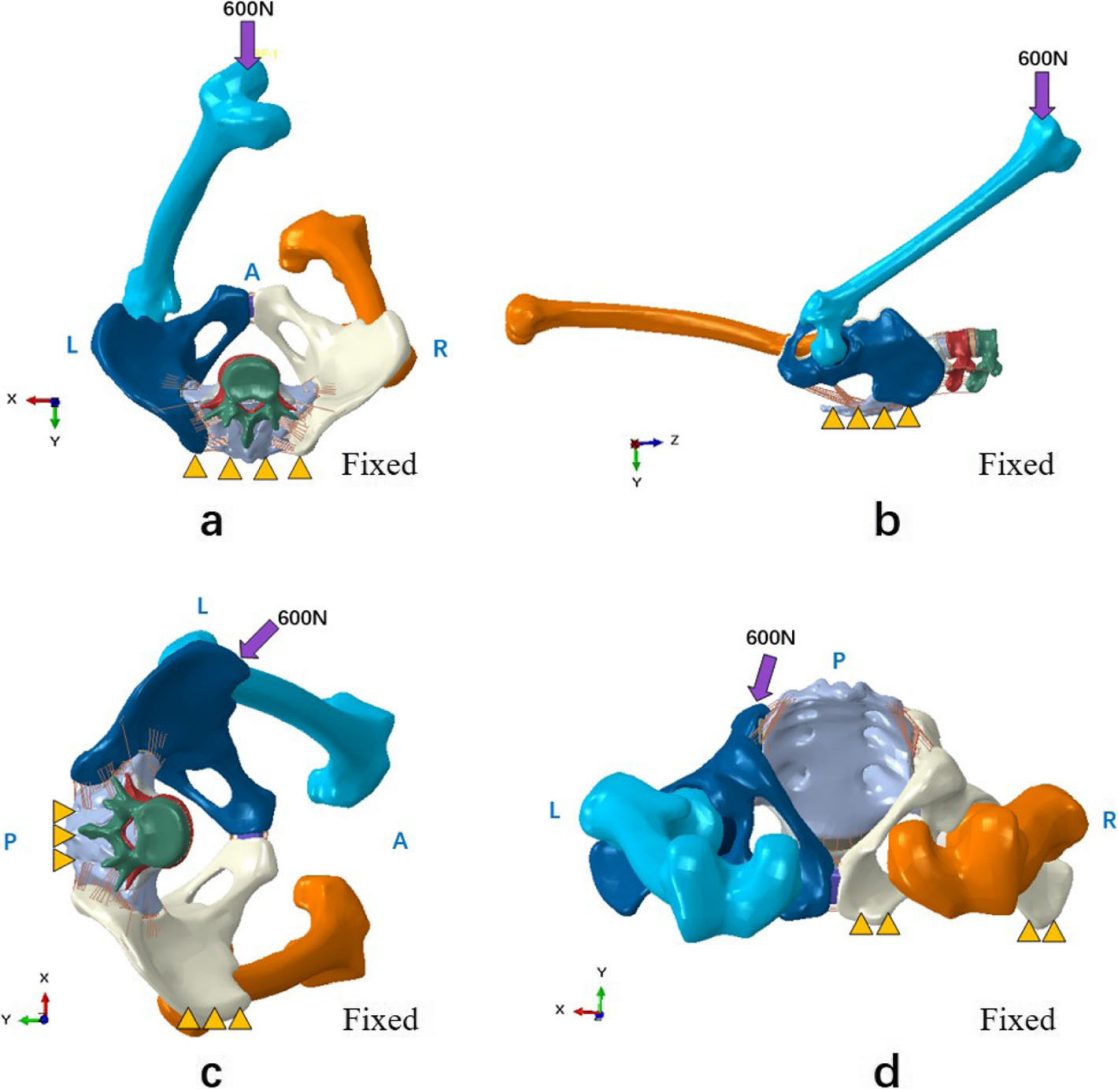
Loading and boundary conditions for the three manipulations. **a** The manipulation of hip and knee flexion (axial view); **b** The manipulation of hip and knee flexion (lateral view); **c** The manipulation of oblique pulling; **d** The manipulation of lower limb hyperextension

Three common manipulations were selected based on their popularity and validity. The point and orientation of the applied forces were determined by previous studies [29, 30]. In addition, the magnitudes of the forces were determined by determining the manipulative power of five therapists using a biomechanical testing machine. The detailed loading and boundary conditions, as well as the x-, y-, and z-axes, are described in Fig. 2. The compressive stresses and displacements of the SIJ and the ligament strains for the three manipulations were then investigated using Abaqus 2018 (Dassault Systemes S. A Company, Massachusetts, USA).

### Manipulation of hip and knee flexion

The patient lay supine while the therapist flexed the patient’s hip and knee as much as possible with pronation. Then, the therapist pushed down the knee, at which point the left hip joint was assumed to be fully constrained. The most posterior regions of the sacrum and the posterior superior iliac spine were fixed. The left hip was flexed to 155° and was intorted to 35°. A compressive (downward) force of 600 N along the ventral-dorsal direction was simultaneously applied at the end of the left femur.

### Manipulation of oblique pulling

The patient was in the side-lying position, and the therapist stood at the patient’s ventral side. The therapist placed one hand on the dorsal side of the sacrum to fix the patient’s position and placed the other hand on the anterior superior spine, pushing the ilium towards the back. Thus, most regions of the sacrum and the right iliac crest were fixed. Then, a push force of 600 N along the ventral-dorsal direction and parallel to the left SIJ surface was applied to the left anterior superior spine.

### Manipulation of lower limb hyperextension

The patient lay in a prone position, and the leg being treated was hyperextended at the hip so that the anterior superior spine could just lift off the bed. Then, the therapist applied a downward force to the iliac crest being treated. In this manner, the right lateral region of the ilium and the right pubic tubercle were fixed. Then, a push force of 600 N along the dorsal-ventral direction and parallel to the left SIJ surface was applied on the left iliac crest. The point of the applied force was the midpoint between the highest point of the iliac crest and the posterior superior spine.

### Mesh convergence study

**Fig. 3:**
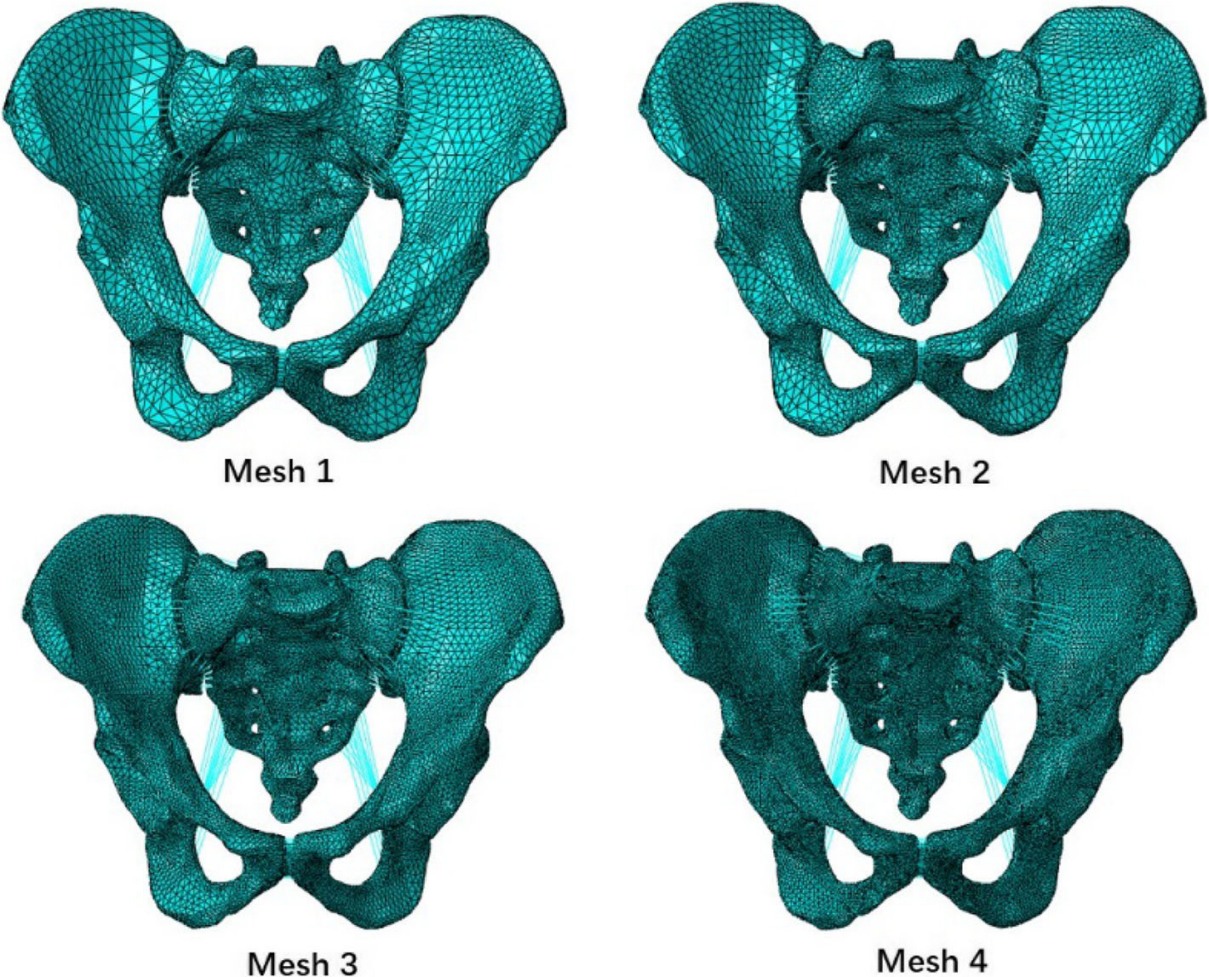
Four meshes for mesh convergence study

To evaluate the degree of accuracy of our FE model, a detailed mesh convergence study was conducted. Four FE models were developed. The number of elements and nodes for each mesh resolution is shown in Table 2. The meshes shown in Fig. 3 were named as mesh 1, mesh 2, mesh 3 and mesh 4, respectively. Following boundary conditions and material properties, loads, and constraints described in detail in the above sections, MHKF, MOP and MLLH were applied to these meshes. The results of the maximum stress and maximum displacement were numerically estimated for each of the meshes.

**Table 2:** Element and node numbers for four different mesh resolutions

	Element number	Node number
Mesh 1	204,097	80,010
Mesh 2	378,211	140,844
Mesh 3	727,474	275,399
Mesh 4	1,603,938	669,044

### Model validation

To validate the developed models, two tests were performed. For the pelvic model, the distribution of the principal strain of the pelvis was compared with that indicated in the study of Zhang [31]. Zhang et al. analyzed the distribution of principal strain on the cortical bone of the pelvis for the single-legged stance. In this model, the distribution of the principal strain of the pelvis was investigated under the same loading and boundary conditions.

For the sacrum model, the relationship between load and displacement was compared to that reported in cadaveric [32] and computational studies [12, 33]. In the cadaveric experiment, the bilateral ilia were fixed. Five translational forces (anterior, posterior, superior, inferior, and mediolateral) of 294 N and three moments (flexion, extension, and axial rotation) of 42 Nm were applied separately to the centre of the sacrum. The displacements of a node lying in the mid-sagittal plane between the inferior S1 and superior S2 vertebral endplates were calculated. In this model, the displacement was estimated under the same loading.

## Results

### Mesh convergence study

**Fig. 4:**
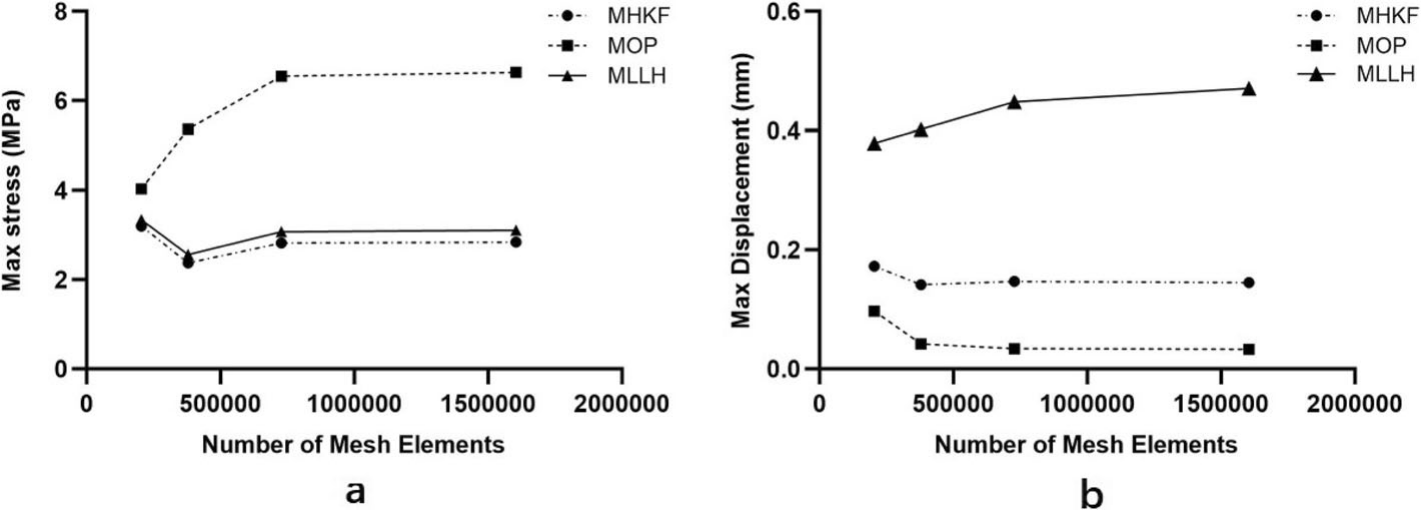
**a** Maximum stress on the left SIJ surface of sacrum for different number of mesh elements, for MHKF, MOP and MLLH. **b** Maximum displacement on the left SIJ surface of sacrum for different number of mesh elements, for MHKF, MOP and MLLH

The results of the maximum stress and maximum displacement on the left SIJ surface of sacrum were investigated for each of the meshes, for MHKF, MOP and MLLH, which can be seen in Fig. 4. The differences in maximum stress and maximum displacement between mesh 3 and mesh 4 in all three manipulations were less than 5%, which was concluded as reasonably close ranges. Based on this finding, mesh 3 with 727,474 elements was selected for further study.

### Model validation

**Fig. 5:**
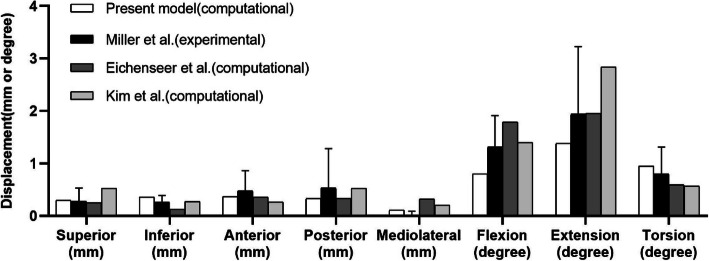
Comparison of sacral displacements under eight loadings comparable to those in previous experimental and computational studies

The principal stresses were distributed mainly in the upper and posterior areas of the acetabulum and extended to the iliac crest, the incisura ischiadica major, and the rear acetabulum. The distribution and maximum value of stress were consistent with those reported in a previous study [31]. Figure 5 shows that the displacements under eight loading conditions were in agreement not only with those in an experimental study but also with those in some computational studies [12, 32, 33].

**Fig. 6:**
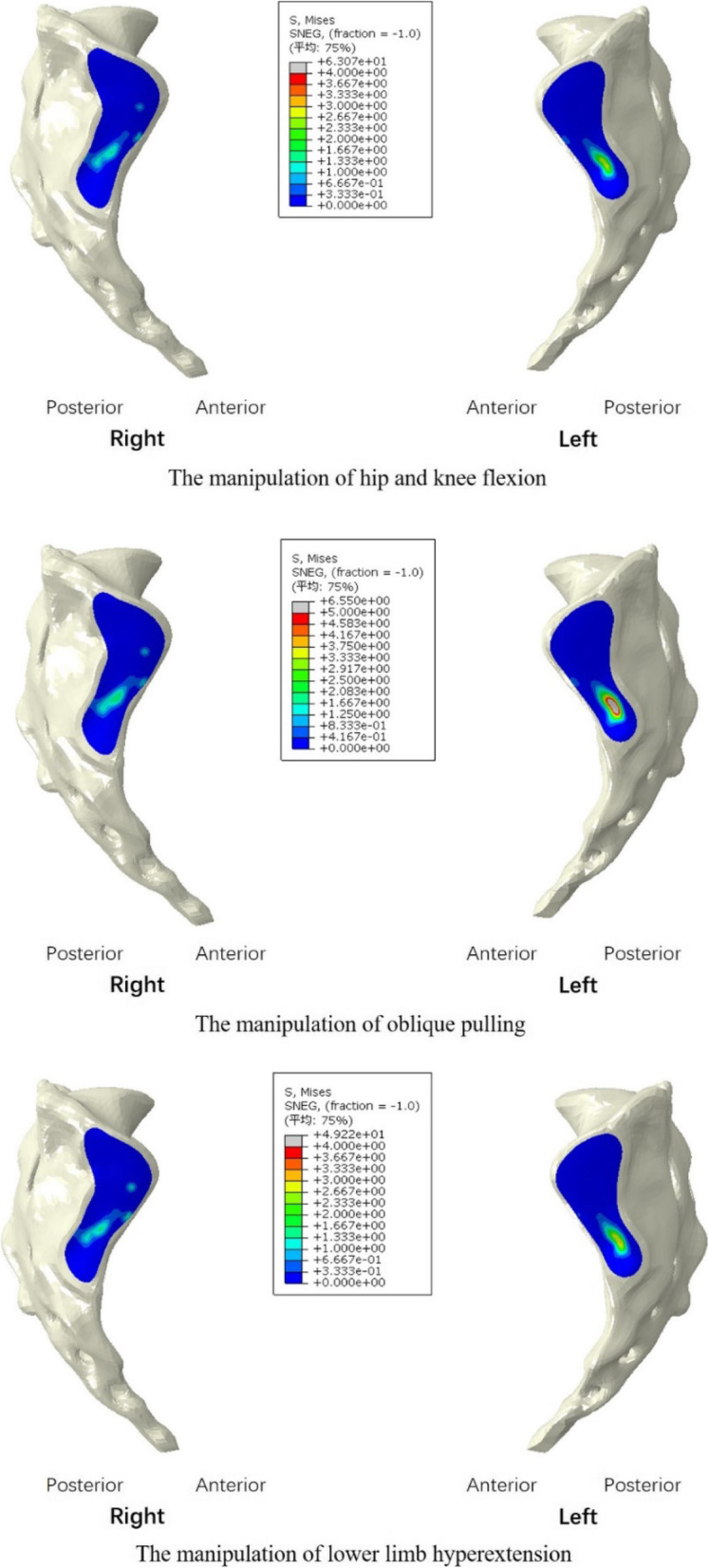
Distribution of compressive stresses on the SIJ surface of sacrum for the three manipulations

The distribution of compressive stresses on the SIJ surface of the sacrum is shown in Fig. 6. Higher stress was observed on the left SIJ for the three manipulations. Among them, MOP could produce the highest stress on the left SIJ, at 6.6 MPa, while MHKF could produce the lowest stress on the right SIJ, at 1.5 MPa.

**Fig. 7:**
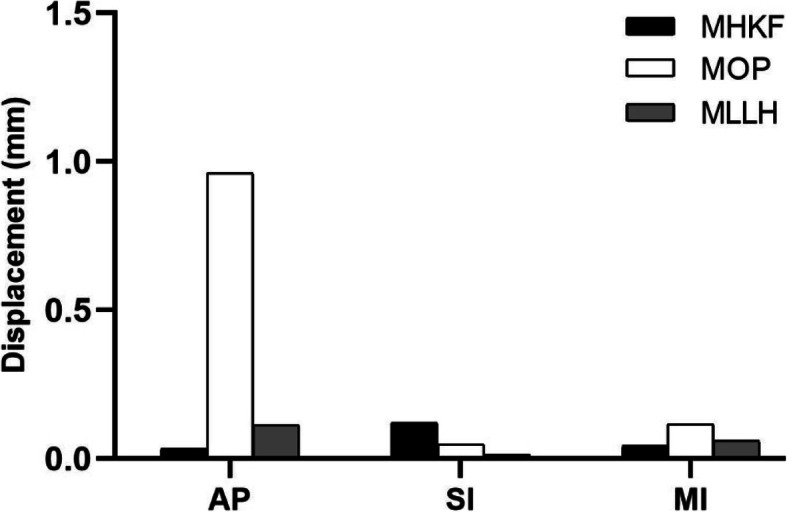
The left sacroiliac joint displacements for the three manipulations. MHKF: The manipulation of hip and knee flexion; MOP: The manipulation of oblique pulling; MLLH: The manipulation of lower limb hyperextension. AP: Anterior-posterior direction; SI: Superior-inferior direction; MI: Medial-lateral direction

In MHKF, the displacements of the left SIJ were 0.120, 0.033, and 0.043 mm in the superior-inferior (SI), anterior-posterior (AP), and medial-lateral (MI) directions, respectively. In MOP, the displacements were 0.048, 0.962, and 0.117 mm in the SI, AP, and MI directions, respectively. In MLLH, the displacements were 0.013, 0.114, and 0.060 mm in the SI, AP, and MI directions, respectively. MOP produced the largest displacement in the AP and MI directions, while MHKF produced the largest displacement in the SI direction. The displacements of the left SIJ are shown in Fig. 7.

**Fig. 8:**
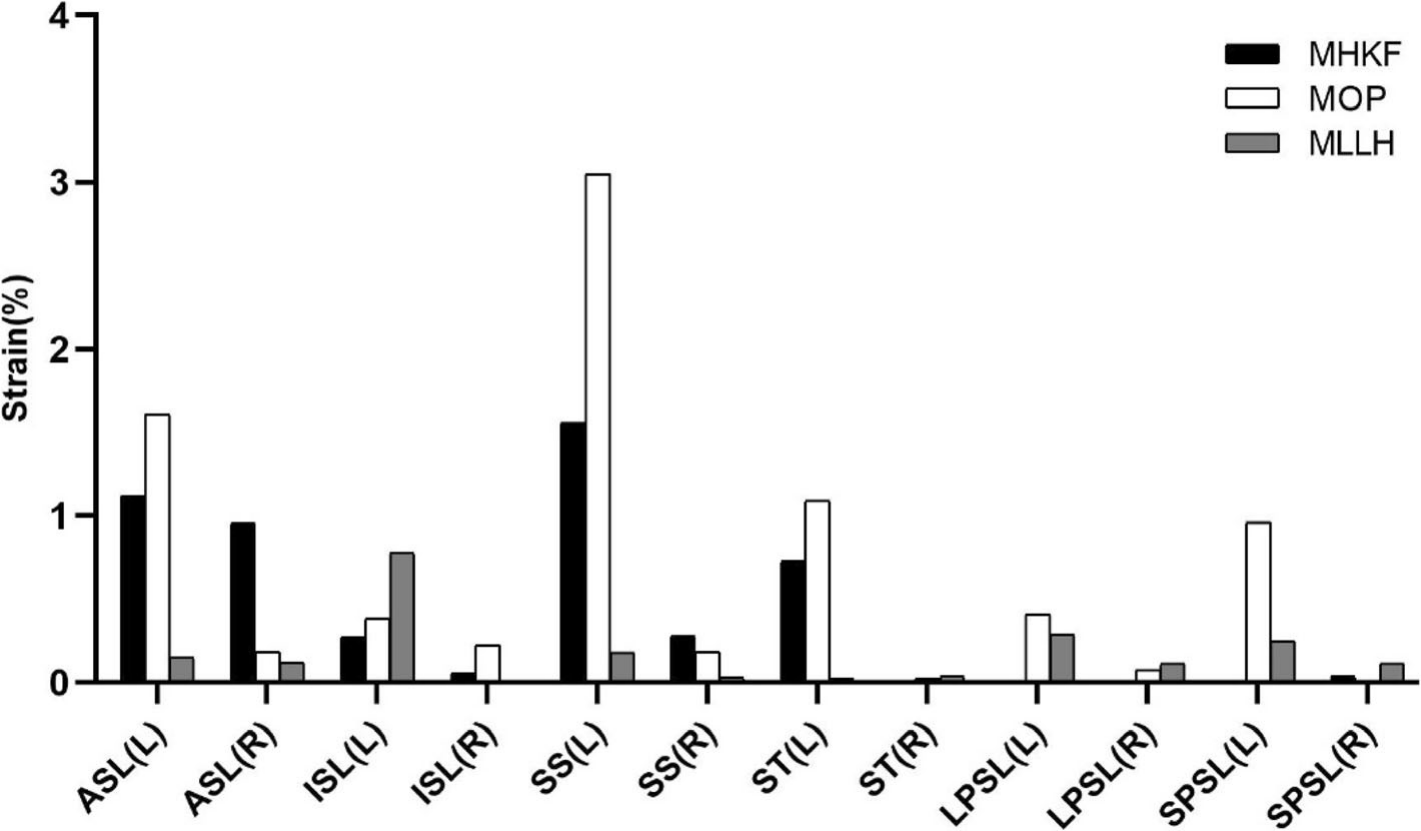
Ligament strains for the three manipulations. MHKF: The manipulation of hip and knee flexion; MOP: The manipulation of oblique pulling; MLLH: The manipulation of lower limb hyperextension; L: Left; R: Right; ASL: Anterior sacroiliac ligament; ISL: Interosseous sacroiliac ligament; SS: Sacrospinous ligament; ST: Sacrotuberous ligament; LPSL: long posterior sacroiliac ligament; SPSL: Short posterior sacroiliac ligament

The strains of six ligaments for the three manipulations are shown in Fig. 8. For most of the ligaments, the strain of the left ligament was greater than that of the right ligament under the three manipulations. In MHKF, the left SS, ASL and ST had the highest strain values, which were 1.6, 1.1 and 0.7%, respectively. MLLH produced the lowest strains of ligaments, while MOP produced the highest strains of ligaments. The left ISL and LPSL had the highest strain values (0.8 and 0.3%, respectively) in MLLH. In MOP, the left SS, ASL, and ST had the greatest strain values, which were 3.1, 1.6, and 1.1%, respectively.

## Discussion

SIJ pain is a common disease that affects 90% of adults throughout their lives [2]. Manipulations have a good effect on SIJ pain with no specific cause. However, the mechanism underlying the effects of manipulations on the SIJ and the ligaments are not yet clear. In this study, a 3D finite element model was used to quantitatively analyze the effects of three manipulations on the displacements of the SIJ and the strains of surrounding ligaments, thus providing a theoretical basis for the indications for manipulations.

In this study, we found that higher stress was observed on the left SIJ, which may be related to the manipulative force applied to the left SIJ. MOP applied force to the iliac directly, and MHKF applied force to the femur indirectly. Therefore, MOP produced the maximum force on the left SIJ, and MHKF produced the minimum force on the right SIJ.

The anterior part of the SIJ is the synovium, which can move slightly, and the posterior part is the interosseous ligament, which mainly plays a role in maintaining the stability of the joint. Walker et al. [34] found that the SIJ had a displacement range of motion of less than 3 mm and a rotation of no more than 2° in a standing or sitting position. Some researchers found that the slip of the SIJ did not even exceed 1 mm [35]. In this study, it was found that MOP could produce the maximum displacement of the SIJ among the three manipulations, with a value of 0.962 mm, while MLLH could cause the minimum displacement of the SIJ, with a value of 0.114 mm. The displacements of the SIJ under the three manipulations were all less than 1 mm, which is consistent with previous studies [34, 35].

In MLLH, both the sacrum and iliac bone were unfixed directly. As a result, they moved simultaneously during the process. Therefore, the relative displacement of the joint surface was small. The displacement produced by MHKF was also small, which might be related to the point of force at the distal femur. The displacement produced by MOP was the largest, considering that the point of force was on the pelvis, and the direction of force was medial-lateral. MOP produced the largest displacement in the AP and MI directions, and MHKF produced the largest displacement in the SI direction. The biomechanical properties of manipulations can provide a theoretical basis for selecting a manual therapy technique.

Ligaments play an important role in maintaining the stability of the pelvis. Sichting et al. [36] found that ligaments serve as the mechanical stabilization device of the pelvis. Bohme et al. [37] observed that the ASL and ST had the greatest load, with 80 and 17% of the total load, respectively, in anteroposterior compression pelvic injuries and that the SS played an important role in the vertical stability of the pelvis. Eichenseer et al. [33] suggested that the ligaments around the SIJ could limit its movement and reduce its stress. This study found that the three manipulations could cause different degrees of strain on the ligaments around the SIJ. In MOP, the patient’s sacrum was relatively fixed, the point of force was on the anterior superior iliac spine, and the force was in the MI direction. Therefore, MOP caused the greatest strain on the ligaments among the three manipulations. The ligament strains produced by MLLH and MHKF were smaller, a finding that might be related to the point and direction of manipulative force as well as the style of pelvic fixation. These results indicate that SS, ASL and ISL experienced the greatest strain under the three manipulations, which was consistent with previous studies [12, 33].

In MLLH, the displacements were the smallest, and the ligament strains were also the smallest. In MOP, the displacements and the ligament strains were both the largest. The displacements of SIJ and the ligament strains remain consistent under the three manipulations. These results also prove the reliability and effectiveness of the model. According to these results, it can be found that MOP is the manipulation that causes the greatest changes in the environment around the SIJ. These results were based on the condition that the manipulations acted on the normal SIJs. However, manipulations were applied on the patients with SIJ subluxation, so these results may have certain errors. In theory, if manipulations can produce the displacement of normal SIJ or the ligament strain, it can also lead to the movement of subluxated SIJ or the change of ligament strain.

Szadek et al. [38] found that there were substance P and calcitonin gene-related peptide-positive nerve fibres in the SIJ cartilage and surrounding ligaments, indicating that the source of SIJ pain might be cartilage and ligament tissues. Is it then possible to reduce pain by pulling the subluxated SIJ back or by alleviating spasms of the surrounding ligaments? Chen et al. [39] suggested that manipulations are unlikely to pull the SIJ back. The clicking sound and the sense of movement during the manipulative process are likely due to movement of attachment of the SIJ or L5/S1 facet joints. Tullberg et al. [26] argued that manipulations cannot change the position of the SIJ and that the pain relief was related to changes in the soft tissues around the joints. Ivanov et al. [40] also suggested that the ligaments around the SIJ contained a considerable amount of nerve tissue and that even a small strain would cause pain. Based on the results of this study, the displacements of the SIJ were less than 1 mm for all three manipulations. In fact, because there are many muscles and other soft tissues around the SIJ in the human body, it can be assumed that any displacement will be small and that it is difficult to pull the SIJ back with manipulations. However, the manipulations did cause different degrees of strain on the surrounding ligaments. Although the degree of ligament strain was small, it could still relieve the spasm of the surrounding ligaments and reduce pain.

There are some limitations of this study. First, our finite element model is based on the geometric and material properties of individual pelvic bones and ligaments in a single male case. However, it is well known that the anatomical structures of the pelvis differ greatly among individuals. This factor must be considered when drawing conclusions in clinical studies. Second, muscles and other soft tissues are most likely to participate in maintaining pelvic stability, and manipulative forces were applied on soft tissues, not directly on bony structures. These factors with muscles and soft tissues were not considered in this model. Third, the ligaments’ characteristics are regarded as linear. Fourth, there is currently no unified standard for manipulations. Therefore, the specific processes of manipulations were simulated and simplified based on the experience of many physicians. Fifth, manipulations are used to treat SIJ subluxation, but the mechanisms of manipulations were investigated with normal SIJ in this study. Therefore, these results may not fully reflect the effect of manipulations. The model of SIJ subluxation and ligament spasm is difficult to establish, and we also plan to do further research in the future.

## Conclusions

This study was the first to analyze the effects of three manipulations on the stresses and displacements of the SIJ and the strains of the surrounding ligaments. The results showed that the displacements of the SIJ produced by the three manipulations were small, but the three manipulations could produce different degrees of ligament strains, which might explain how the manipulations relieve SIJ pain. MOP produced the largest displacement and the greatest ligament strains.

## Data Availability

The datasets used and/or analyzed during the current study are available from the corresponding author upon reasonable request.

## Abbreviations

SIJ: Sacroiliac joint
MHKF: The manipulation of hip and knee flexion
MOP: The manipulation of oblique pulling
MLLH: The manipulation of lower limb hyperextension
ASL: Anterior sacroiliac ligament
ISL: Interosseous sacroiliac ligament
SS: Sacrospinous ligament
ST: Sacrotuberous ligament
LPSL: Long posterior sacroiliac ligament
SPSL: Short posterior sacroiliac ligament
AP: Anterior-posterior direction
SI: Superior-inferior direction
MI: Medial-lateral direction
